# Efficiency of a Tetracycline-Adjuvant Combination Against Multidrug Resistant *Pseudomonas aeruginosa* Tunisian Clinical Isolates

**DOI:** 10.3390/antibiotics9120919

**Published:** 2020-12-17

**Authors:** Azza Troudi, Meha Fethi, Mohamed Selim El Asli, Jean Michel Bolla, Naouel Klibi, Jean Michel Brunel

**Affiliations:** 1 Faculté de Pharmacie, Aix Marseille Universite, INSERM, SSA, MCT, 13385 Marseille, France; azzatroudi.92@gmail.com (A.T.); jean-michel.bolla@univ-amu.fr (J.M.B.); 2 Laboratory of Microorganisms and Active Biomolecules, Department of Biology, Faculty of Sciences of Tunis, University of Tunis, Tunis 1008, Tunisia; meha.fethi@yahoo.fr (M.F.); n_klibi@yahoo.fr (N.K.); 3 Service of Microbiology, Military Hospital of Tunis HMPIT, Tunis 1008, Tunisia; elasliselim@yahoo.fr

**Keywords:** polyaminoisoprenyl derivatives, antibiotic adjuvants, polyamines, *Pseudomonas aeruginosa* clinical strains, doxycycline, minocycline

## Abstract

The growing number of multidrug resistant strains in Tunisia has become a serious health concern contributing to high rate of mortality and morbidity. Since current antibiotics are rapidly becoming ineffective, novel strategies to combat resistance are needed. Recently, we demonstrated that combination of a tetracycline antibiotic with various polyaminoisoprenyl adjuvants can sustain the life span and enhance the activity of these drugs against *Pseudomonas aeruginosa* reference strain (PA01). In the context of our continuing studies, the effective approach of antibiotic-adjuvant was investigated against a large panel of *P. aeruginosa* Tunisian clinical strains collected from the Military Hospital of Tunis. In this paper, we demonstrated that the combination of a farnesyl spermine compound **3** used at concentrations ranging from 2.5 to 10 µM, in the presence of doxycycline or minocycline leads to a significant decrease of *P. aeruginosa* antibiotic resistance.

## 1. Introduction

Antibiotics are considered probably as one of the most successful drug form in medicine history [1]. They saved countless lives and placed the infectious diseases under control for several years. The World Health Organization (WHO) showed a decrease in these infection rates over the 20th century varying from almost half of total death to less than 10% [2]. Nevertheless, since their discovery, it clearly appeared that antimicrobial drugs have gradually lost their efficiency due the emergence and the spread of antibiotic resistant strains especially multidrug-resistant bacteria (MDR) which appear now as among the major health threats for humans [3,4]. In this context, *Pseudomonas aeruginosa* is one of the most common Gram-negative bacteria associated with nosocomial infections especially in the intensive care units (ICUs) as well as for serious infections in cystic fibrosis patients [5], severe burns [6], as well as immunocompromised patients [7]. This species is intrinsically resistant to several antibiotic classes such as tetracyclines which limits the therapeutic options. Between 2009 and 2014 study in Tunisia showed that nosocomial infectious in ICU are majorly caused by a high prevalence rate of *P. aeruginosa* which represents 22.86% from the total infections [8]. The increasing frequency of multidrug-resistant *P. aeruginosa* (MDRPA) in Tunisia becomes a serious health public concern especially imipenem-resistant *P. aeruginosa* isolates. The most frequent mechanisms of resistance encountered deal with beta-lactamase production (carbapenemases), modification of porin OprD involved in outer membrane permeability, and overexpression of active efflux pumps [9]. Thus, the WHO confirmed that “without urgent action, the world is headed for a post-antibiotic era, in which common infections and minor injuries which have been treatable for decades can once again kill” [10]. Otherwise, to avoid the therapeutic impasse, creative approaches of prevention and control are self-evident to combat resistance [11,12]. In this context, we recently demonstrated that an antibiotic-adjuvant approach based on the use of lowly cytotoxic polyaminoisoprenyl derivatives and tetracycline antibiotics in combination represents a novel efficient strategy to restore the activity of these drugs and increase the susceptibility of a *P. aeruginosa* reference strains.

The aim of this study was to investigate the efficiency of our approach against many clinical strains collected from Tunisian hospital and to establish a close resistance profile–activity relationship.

## 2. Experimental Section

### 2.1. Synthesis of Compound **3**

To a solution of spermine (450 mg, 2.27 mmol) and triethylamine (450 µL, 4.5 mmol) in distillated Tetrahydrofurane (THF) (10 mL) was added dropwise farnesyl chloride **1** (480 mg, 2 mmol) in distillated THF (15 mL). The reaction mixture was stirred at room temperature for 24 h and evaporated to dryness. The crude residue was purified by column chromatography (eluant CH_2_Cl_2_/MeOH/conc. NH_4_OH, 7:3:1) to afford the pure desired compound as a yellow solid in 64% yield. Yellow solid; ^1^H NMR (MeOD, 250 MHz): *δ* = 5.05–4.93 (m, 3H), 2.93–2.57 (m, 14H), 2.19–1.92 (m, 10H), 1.63–1.87 (m, 23H). ^13^C (MeOD): *δ* = 142.24, 134.83, 131.09, 124.86, 124.17, 117.32, 47.90, 47.69, 47.64, 46.61, 44.01, 40.60, 39.83, 33.97, 31.20, 26.97, 26.30, 25.66, 25.11, 24.90, 17.62, 16.90, 15.93. C_25_H_50_N_4_ MS (ESI+) m/z 407.41 (100%, [M + H]^+^) (Figures S1 and S2).

### 2.2. Bacterial Strains

Twenty-one nonduplicated clinical *P. aeruginosa* isolates were recovered from different patients of the Military Hospital of Tunis during October 2018-July 2019 and were isolated from blood (*n* = 5), catheters (*n* = 2), pus (*n* = 3), urine (*n* = 1), tracheal aspirate fluid (*n* = 9), and other origins (*n* = 2). All isolates were identified by Vitek 2 Compact (BioMérieux, Lyon, France) and confirmed by PCR targeting the *opr*L gene [13]. Three reference *P. aeruginosa* strains (PA01, PA7, and PA14) were used in this study [14,15]. These strains were stored in 15% (*v*/*v*) glycerol at −80 °C for cryo-protection and subcultured in Brain Heart Infusion (BHI) agar at 37 °C for inoculum preparation.

### 2.3. Susceptibility Testing

Antibiotic susceptibility testing was performed by the disk diffusion method on Mueller–Hinton agar plates to 13 antibiotic discs (BioRad, Marne-la-Coquette, France): cefepime (FEP), ceftazidime (CAZ), imipenem (IPM), meropenem(MEM), aztreonam (ATM), piperacillin (PIP), piperacillin-tazobactam (PIT), ticarcillin-acid clavulanic (TCC), gentamicin (GEN), amikacin (AMK), tobramycin (TM), ciprofloxacin (CIP), levofloxacin (LVX). From a fresh culture, a bacterial suspension with a concentration of 0.5 Mc Farland was prepared. Then, the Muller–Hinton agar surface was completely seeded by swabbing and the antibiotic discs were placed. Results were interpreted after incubation at 37 °C for 18–24 h based on the guidelines of the European committee on Antimicrobial Susceptibility testing (EUCAST, 2020).

### 2.4. Detection of Efflux Pump Activity

Efflux pumps overexpression was studied using imipenem, meropenem, and ciprofloxacin discs on plates in the presence or absence of the inhibitor Phe-Arg-β-naphthylamide (PAβN, 40 mg/L). Isolates were defined as efflux pump overproducers when more than a 5 mm difference between the antibiotic inhibition zone was encountered in the presence or absence of cloxacillin or PAβN, respectively [16].

### 2.5. MIC Determination of Doxycycline, Minocycline, and Compound **3**

The minimal inhibitory concentration (MIC) is defined as the lowest concentration of an antibiotic which prevents visible growth of a bacterium. Susceptibilities to doxycycline (Sigma, St Quentin-Fallavier, France), minocycline (Sigma), and compound **3** were determined in sterile 96-well microplates by using the standard broth dilution method in accordance with the recommendations of the Comité de l’Antibiogramme de la Société Française de Microbiologie (CA-SFM) [17]. The stock solutions of tetracyclines at 6.4 mg/mL were freshly prepared for each experiment in water or dimethyl sulfoxide (DMSO) as indicated. Briefly, the MICs were determined with an inoculum of 10^5^ CFU in 200 µL of MH broth containing two-fold serial dilutions ranging from 128 to 0.25 µg/mL of each molecule in 96-well plates. The MIC was defined as the lowest concentration of drug that completely inhibited visible growth after incubation for 18 h at 37 °C. All MIC determinations were repeated in triplicate in independent experiments. PA01, PA7, and PA14 were used as reference strains [17,18].

### 2.6. Drug–Drug Interaction Assay

**Figure 1:**
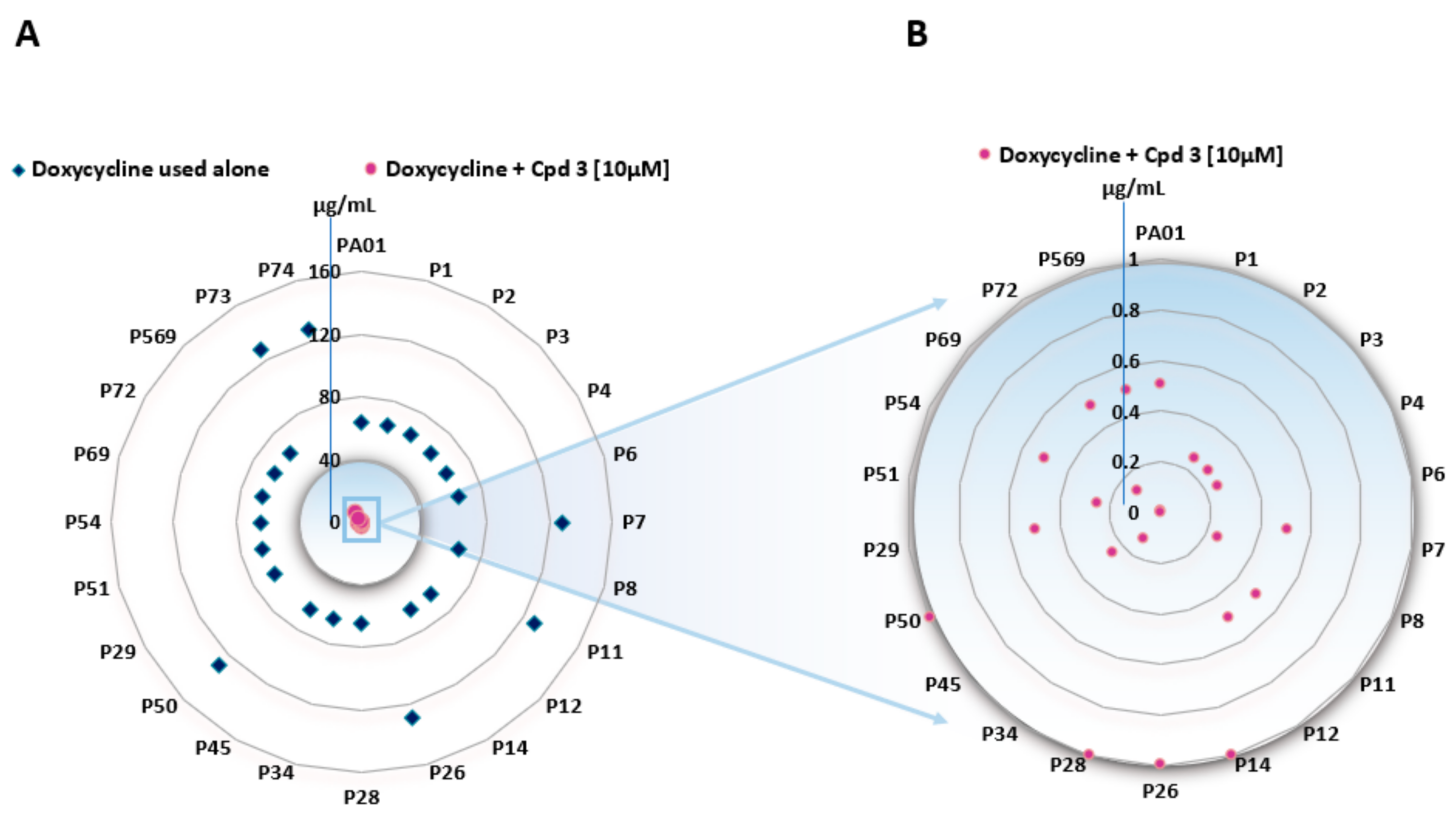
MIC (µg/mL) of doxycycline against PA01 and *P. aeruginosa* Tunisian clinical strains. (**A**) In the absence and (**B**) in combination with compound **3**.

The antimicrobial effects of doxycycline or minocycline combinations with compound **3** were evaluated in sterile 96-well microplates in two ways. In a first approach, the influence of the adjuvant at three different concentrations (2.5, 5, and 10 μM/well) were evaluated on the MIC of doxycycline. A series of cascade dilutions of doxycycline (from 128 to 0.25 μg/mL) was performed from the starting solution (6.4 mg/mL). In each column of the microplate the concentration of adjuvant **3** was set at 2.5, 5, or 10 μM/well. In a second way, doxycycline concentration in each well was fixed at 2 µg/mL which corresponds to the doxycycline susceptibility threshold according to the CLSI (Clinical and Laboratory Standards Institute) (https://clsi.org/meetings/microbiology/), in order to determine the lowest concentration of adjuvant capable of decreasing the MIC of doxycycline at this sensitivity threshold. For both tests, the bacterial suspension was prepared from colonies grown overnight. The concentration was adjusted to 1.5 × 10^5^ CFU/well. The MIC of each combination was determined after 18 h of incubation at 37 °C. All MIC determinations were repeated at least three times in independent experiments. Considering two compounds A and B, the fractional inhibitory concentration index (FICI) was calculated using the following formula: FICI = (MICAcombi/MICAalone) + (MICBcombi/MICBalone) and ranging from 0.41 to 0.46 for all the considered strains which stands for synergy was calculated but the MIC ratio, defined as the ratio of the MIC of each tetracycline antibiotic to its MIC determined in the presence of compound **3** at three different concentrations (2.5, 5, and 10 μM/well), was preferred to present the results (Figure 1).

### 2.7. Outer Membrane Permeabilization Assay 

Nitrocefin was used as a chromogenic substrate of periplasmic β-lactamase to measure the outer membrane permeabilization. The nitrocefin hydrolysis assay is a colometric assay wherein a color change from yellow to red occurs when the chromogenic β-lactam is efficiently hydrolyzed by periplasmic β-lactamases. After an overnight culture of P1 and P73 at 37 °C, 100 µL of each suspension was added to 10 mL of MHII broth. Once the cultures reached the mid-logarithmic phase (OD_600_ = 0.5), cells were recovered by centrifugation (3600× *g* for 20 min at 20 °C) and washed twice with 20 mM potassium phosphate buffer (pH 7.2) and 1 mM MgCl_2_ (PPB). After the second centrifugation, the pellet was resuspended and adjusted at OD_600_ 0.375. Then, 100 µL of each bacterial suspension was mixed with 50 µL of compound **3** at different concentrations ranging from 128 to 8 µM already set up in a 96-well microplate. Polymyxin B (PMB) and polymyxin Nona (PMBn) were used as positive controls and PPB was used as a negative one. Finally, 50 µL of nitrocefin was added to obtain a final concentration of 50 µg/mL. Nitrocefin hydrolysis was monitored by measuring the increase in absorbance at 490 nm, using a M200 Pro Tecan spectrophotometer, for 1 h with a 1-min interval between each measurement. Experiments were performed in triplicate [19,20].

### 2.8. Membrane Depolarization

P1 and P 73 strains were grown in MH II broth for 24 h at 37 °C. After reaching an OD_600 nm_ of 0.5, cells were centrifuged (3600× *g* for 20 min at 20 °C) and washed twice with buffered sucrose solution (250 mM), magnesium sulfate solution (25 mM), and Hepes (5 mM) (pH = 7.2). The fluorescent dye 3,3′-diethylthiacarbocyanine iodide DiSC_3_(5) was added to a final concentration of 5 µM and was incubated with the suspensions for 5 min at 37 °C to allow the dye incorporation into the polarized membranes. Then, 10 µL of compound **3** was added to 90 µL of the fluorescent suspensions at different concentrations ranging from 250 to 7.8 µM. Fluorescence measurements were recorded after 1, 5, 10, and 15 min (excitation wavelength 622 nm, emission wavelength 690 nm) [19].

The difference in the relative fluorescence values (RFU) from the control containing only buffer and the control containing bacteria treated only with cetyltrimethylammonium bromide (CTAB 1%) was taken as the maximum level of depolarization. Assays were performed in three independent experiments. 

Equation used

$$\frac{\left(\mathrm{RFU}\;\mathrm{of}\;\mathrm{the}\;\mathrm{compound}-\mathrm{RFU}\;\mathrm{of}\;\mathrm{the}\;\mathrm{blank}\right)\;*\;100}{\mathrm{RFU}\;\mathrm{of}\;\mathrm{the}\;\mathrm{maximum}\;}$$

Blank determined by using no compound.

Maximum RFU determined by using CTAB 0.1%.

### 2.9. Glucose-Triggered 1,2′-diNA Efflux Assays

Bacteria were grown until the stationary phase was reached, collected by centrifugation, and resuspended at OD_600 nm_ of 0.25 in PPB supplemented with carbonyl cyanide m-chlorophenyl hydrazone (CCCP, 5 µM), and incubated overnight with 1,2′-dinaphthylamine (1,2′-diNA, 32 µM) at 37 °C. Before addition of the desired compound at different concentration ranging from 250 to 7.8 µM, the cells were washed with potassium phosphate buffer (PPB). Cell suspension was added at 100 µL per well and the fluorescence was read every 30 s at 37 °C. Glucose (50 mm) was added after 300 s to initiate bacterial energization. Membrane-incorporated 1,2′-diNA was observed by monitoring the fluorescence (lex = 370 nm; lem = 420 nm). An Infinite M200Pro reader (Tecan) was used. Assays were performed in Greiner Bio-One 96-well plates (ref. 675076; half area, black with clear bottom).

### 2.10. Statistical Analysis

Data were analyzed using Student’s *t*-test analysis for differences between two groups, and findings were expressed as mean + SD. All assays included two replicates and were repeated in at least two independent experiments. A *p* value < 0.05 was statistically significant.

## 3. Results

### 3.1. Synthesis of Polyaminofarnesyl Derivative **3**

**Scheme 1:**
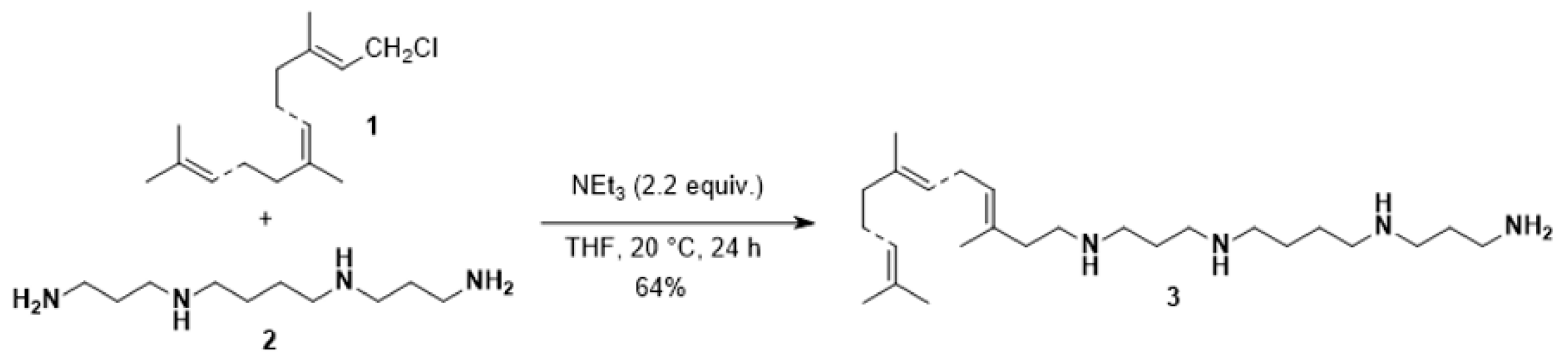
Synthesis of polyaminofarnesyl derivative **3.**

According to our previously reported studies, we chose to focus our attention on the synthesis of derivative **3.** Thus, according to the following synthetic pathway the expected compound in 64% isolated yield (Scheme 1).

### 3.2. Characteristics of the Bacterial Strains and MIC Determination

Our study involved 21 *P. aeruginosa* strains collected from patients hospitalized in the Military Hospital of Tunis. All isolates showed resistance to imipenem and meropenem and are multidrug resistant, 16 of them being resistant to more than 11 different antibiotics (Table S1).

We first evaluated the MICs of compound **3**, doxycycline, and minocycline against a large panel of the Tunisian *P. aeruginosa* clinical strains allowing us to determine the amount of **3** that could be added to each strain without producing a direct antibacterial activity. As summarized in Table 1, our experiment revealed that MICs values of compound **3** were ranging from 12.5 to 50 µM whereas those of doxycycline and minocycline were from 64 to 128 µg/mL and 32 to 128 µg/mL, respectively (Table 1) (for all results see Table S2).

Compound **3** was then assayed for its ability to potentiate doxycycline and minocycline activity against the large panel of *P. aeruginosa* Tunisian clinical isolates at three different fixed concentrations (2.5, 5, 10 µM). In a first approach, the MICs values encountered for doxycycline in the presence of compound **3** used at a 10 μM concentration ranged from 0.005 to 1 µg/mL for all the bacterial strains tested (Figure 1B and Table S3) except for the strains P73 and P74 which are the most resistant with MICs ranging from 8 to 4 µg/mL, respectively.

**Figure 2:**
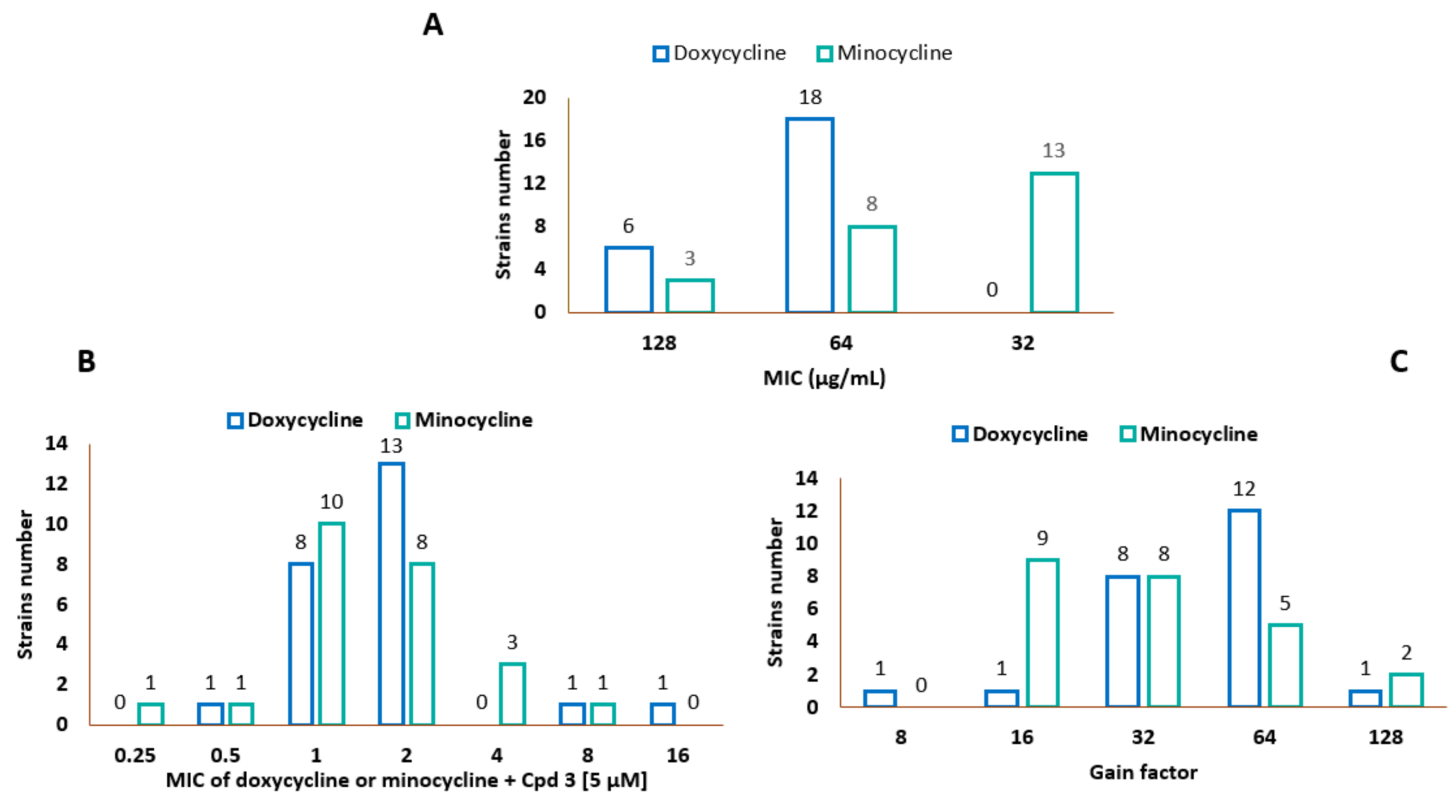
Comparison of *P. aeruginosa* strains number obtained in MIC assay with doxycycline and minocycline (**A**), their combination with compound **3** at 5 µM (**B**), and the encountered gain factor (**C**).

In further experiments, a comparison of the ability of compound **3** to enhance the activity of doxycycline to minocycline, was tested at three different concentrations (2.5, 5, 10 µM) against a selection of *P. aeruginosa* PA01 and Tunisian clinical strains (Figure 2). All the strains demonstrated a poor susceptibility towards both antibiotics (Figure 2A). Otherwise, polyaminoisoprenyl derivative **3** exhibited a high efficiency for increasing minocycline susceptibility of 87% of the strains with a gain factor ranging from 32 to 64-fold when used at a 10 μM concentration (Figure 2B) (the gain factor corresponding to the ratio of the MIC of the antibiotic tested alone to the MIC of the antibiotic obtained in the presence of the considered adjuvant). From this experiment we observed that the strains P1 and P6 appeared as the most susceptible ones whereas both strains P73 and P74 were the most resistant towards the action of the combination.

**Figure 3:**
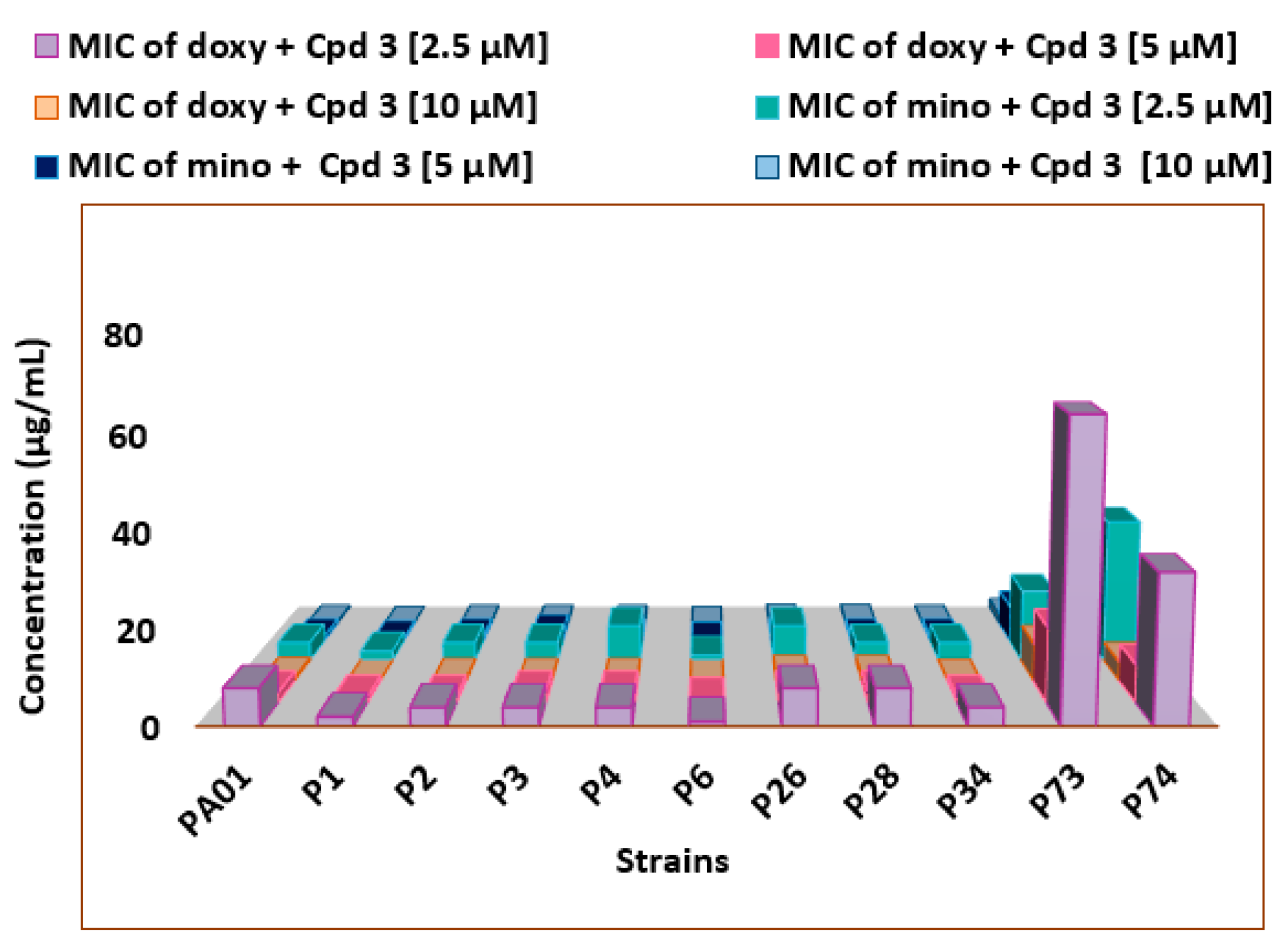
Dose-dependent effect of the compound **3** in combination with doxycycline (doxy) and minocycline (mino) against *P. aeruginosa* clinical strains.

Comparatively, P73 and P74 were found to be the least responsive to the action of compound 3 (10 µM) with the highest MIC values obtained for minocycline (2 and 1 µg/mL) and doxycycline (8 and 4 µg/mL), respectively (Figure 3). In all the other cases, the compound **3** was found to be extremely active at 10 µM and the synergistic effect involving **3** and minocycline led to moderate to excellent results with a gain factor ranging from 32 to up to 512-fold depending the considered experimental conditions. Altogether these results demonstrated an adjuvant dose-dependent potentiation of the tetracyclines activity.

### 3.3. Mechanism of Action

Based on the encountered biological activities, two susceptible strains P1 and P6 and two resistant ones P74 and P73 have drawn our attention. Thus, in order to understand more precisely the mechanism of action of 3, two representative strains were selected (P1 and P73), to evaluate the potent permeabilizing, depolarizing, and disrupting behavior of compound **3** on the outer and/or inner membranes of these two strains. We attempted also to determine its ability to act as an efflux pump inhibitor.

#### 3.3.1. Outer Membrane Permeabilization

**Figure 4:**
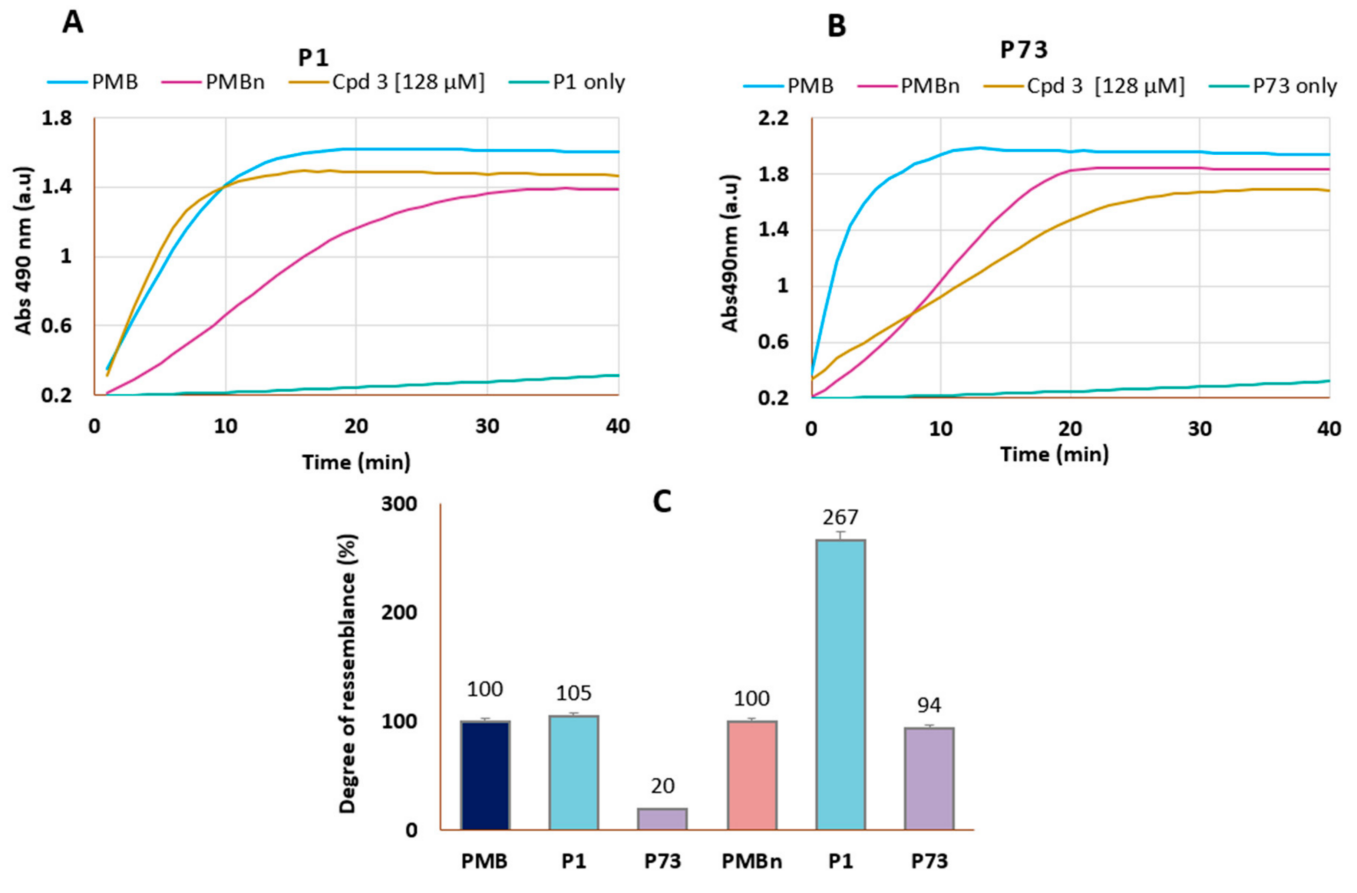
Study of outer membrane permeabilization of P1 (**A**) and P73 (**B**) by evaluating the rate of nitrocefin hydrolysis in the presence of PMB (200 µM), PMBn (106.3 µM), and compound **3** at 128 µM. Membrane permeation effects of compound **3** at 128 µM compared to PMB and PMBn (**C**)**.**

As previously reported, the permeabilization activity of the compound **3** and its capacity to disrupt the integrity of the outer membrane was evaluated by using the nitrocefin hydrolysis assay [21,22,23]. Thus, permeation of nitrocefin was measured to determine if compound **3** exhibited a difference in the outer membrane permeability between the two isolates producing the β-lactamases. The curves of nitrocefin hydrolysis kinetics of P1 and P73 in the presence of a high concentration of compound **3** (128 µg/mL) compared to those obtained for the two positive controls (PMB and PMBn) are presented in Figure 4A,B.

Interestingly, **3** increased the rate of the nitrocefin hydrolysis differently depending on the considered strain suggesting that the behavior of this compound with respect to the nature of the outer membrane of P1 and P73 is not identical. Thus, compound **3** is able to disrupt the outer membrane of P1 by acting in a similar manner to PMB (Figure 4A) whereas it exhibited a permeabilizing activity close to that of PMBn against P73 (Figure 4B). The degree of resemblance percentage was also calculated to evaluate the potency of the compound 3 to permeabilize the outer membrane compared to that of PMB or PMBn (Figure 4C) demonstrating that 105% of similarity of compound 3 compared to PMB in P1 was encountered whereas it was only 20% in the case of P73. Altogether, these data are in a perfect correlation with those obtained in the antibiotic-adjuvant MIC test (Table 1) confirming the similar behavior of compound **3** to PMB and PMBn against P1 and P73, respectively.

#### 3.3.2. Membrane Depolarization Assay

**Figure 5:**
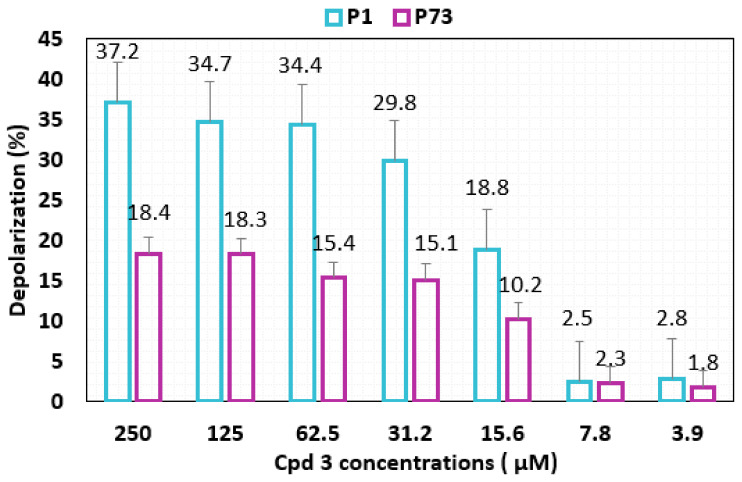
Study of the inner membrane perturbation of P1 and P73 by evaluating DiSC_3_(5) fluorescence recorded after 5 min in the presence of compound **3** at different concentrations.

To determine the capacity of compound **3** to affect the inner membrane, the membrane potential sensitive probe DiSC_3_(5) [24] was used and concentrated at the inner membrane level of the selected strains P1 and P73 (Figure 5). The dye was released when the tested compound alters the inner membrane, leading to an increase in fluorescence intensity. As shown in Figure 5, compound **3** induced an increase of the level of fluorescence in P1 and P73 which is related to the increase of the inner membrane depolarization. Interestingly the level of depolarization in the strain P1 was almost 2-fold compared to that of P73 whatever the concentrations tested.

#### 3.3.3. Glucose-Triggered 1,2′-diNA Efflux Assays

In a third attempt, we investigated the ability of compound **3** to act as an inhibitor of efflux pumps. In this context, strains P1 and P73 were loaded with 1,2′-diNA dye which is a substrate of the *P. aeruginosa* efflux pumps [25]. Glucose was then added as the energy source after incubation of the two strains in the presence of compound **3** at different concentrations ranging from 250 to 7.8 µM.

**Figure 6:**
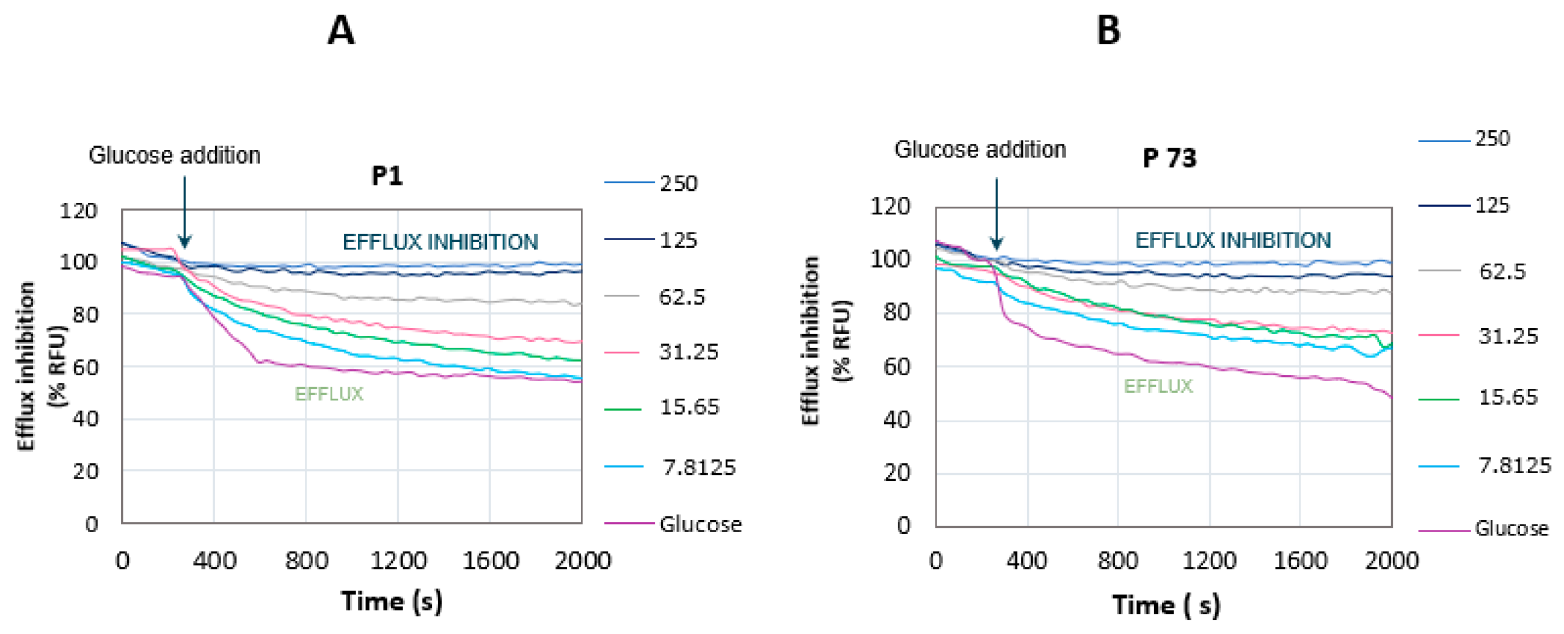
Inhibition of the efflux of 1,2′-dinaphthylamine by compound **3** at different concentrations against P1 and P73. Efflux was triggered after 300 s by addition of glucose.

The highest rate of the active transport of the dye is observed for bacteria untreated and the two strains were able to both expel the dye (Figure 6A,B red curve). Interestingly, **3** demonstrated efflux inhibition in a significant dose dependent manner. At high concentration (250 to 62.5 µM) a strong inhibition is observed resulting in up to 90% of dye retention for the two strains whereas at the lowest concentrations the curves appeared like that of untreated bacteria.

## 4. Discussion

This study included 21 *P. aeruginosa* strains resistant to carbapenems isolated from the Military hospital of Tunis [12,13]. All the considered isolates are multiresistant to different antibiotics in addition to their natural resistance to tetracyclines. Commonly, Gram-negative bacteria proved to be the less sensitive strains, being resistant to many classes of antibiotics due to their outer lipidic membrane that restricts the access to the periplasm by acting as an efficient selective permeation barrier. Recently, we reported a great synergistic activity of the doxycycline–compound **3** combination against PA01 reference strain with an increase in susceptibility from 128 to 0.5 µg/mL with a gain factor of 128-fold when compound **3** was used at a 10 μM concentration. Better results were also obtained by using minocycline instead of doxycycline which could be due to its higher hydrophobicity increasing its affinity to interact with the destabilized outer membrane to penetrate the *P. aeruginosa* bacteria. Based on our results, it appeared that there is no significant relationship between the resistance phenotypes of clinical *P. aeruginosa* strains and the MICs encountered for doxycycline and minocycline used in combination with polyaminoisoprenyl compound **3** against these isolates. The strains P1 and P6 were found the most sensitive in the presence of the tetracyclines/compound **3** combination and do not present any difference in profile and resistance genes even if they are genetically different by pulsed field electrophoresis and multilocus sequence typing. Nevertheless, it is noteworthy that contrarily to all the other strains, P1 and P6 do not exhibit efflux pump overexpression which could explain the low MICs encountered since it is well admitted that efflux pumps may represent the main mechanism of resistance to cyclins. On the other hand, the P73 and P74 strains, belonging to the ST235-O11clone [26] showed a multidrug resistance and virulence genotype in hospitals especially in Mediterranean countries (unpublished data), overexpress efflux pumps, and appeared to be the most resistant towards the tetracycline/compound **3** combination [27]. On the other hand, strains P29 and P69 also showed increased sensitivity compared to other genetically different strains.

Since we determined that compound **3** strongly decreases the MIC of tetracyclines family against *P. aeruginosa*, it was necessary to investigate the mechanism of action of this compound. That was based on the study of the chromogenic β-lactam nitrocefin hydrolysis by periplasmic beta-lactamases leading to a change in color from yellow to red and directly related to the integrity of the outer membrane. All our data suggest that compound **3** disrupts the outer membrane integrity of Gram-negative bacteria in a dose-dependent manner as already observed for compound **3** parent derivatives against *B. bronchiseptica*, *E. aerogenes*, and *S. enterica* [19,28,29]. This study also demonstrated that **3** could also depolarize the inner membrane in a dose-dependent manner and interestingly, this depolarization was two times higher in the case of P1 than P73. Both differences observed between these two strains in the nitrocefin and the depolarization assays lead to the conclusion that compound **3** does not act in similar manner against these two strains and that this behavior could be related to their different membranes composition [30]. Moreover, the profile of the efflux pumps inhibition assay demonstrates that the compound **3** does not exhibit high affinity for the pump site at low concentration (<62.5 µM). The inhibition obtained in the two strains was observed with a clear dose–response effect. The same result was obtained for several farnesyl compounds as encountered in a previous work on *Pseudomonas* [25,31].

Interestingly, no differences were noticed by comparing the profiles of P1 which do not exhibit efflux pump overexpression and P73 which overexpress the efflux pumps [32]. Nevertheless, one cannot exclude that at high concentration, such an inhibition of the efflux pump may result from various actions including either direct interaction with the pump by blocking the antibiotic transfer or competing with the antibiotic during its transfer and by energy disruption or by inhibition of the pump assembly [33]. Finally, compound **3** remains of interest for a therapeutic human use development since we have previously determined that it had low cytotoxicity with an IC50 against CHO cell line of 142 µM [33].

## Figures and Tables

**Table 1:** MICs of doxycycline, minocycline, and compound **3** against PA01 and selected *Pseudomonas aeruginosa* Tunisian clinical strains.

Entry	Strains	MIC of Doxycycline (μg/mL)	MIC of Minocycline (μg/mL)	MIC of Compound 3 (µg/mL) (μM)
1	PA01	64	32	10 (25)
2	P1	64	32	10 (25)
3	P2	64	32	10 (25)
4	P3	64	32	10 (25)
5	P4	64	32	10 (25)
6	P6	64	32	5 (12.5)
7	P26	128	128	10 (25)
8	P28	64	64	10 (25)
9	P29	64	64	10 (25)
10	P34	64	64	10 (25)
11	P73	128	128	10 (25)
12	P74	128	64	20 (50)

## Supplementary Materials

The following are available online at https://www.mdpi.com/2079-6382/9/12/919/s1, Table S1: Origins, antimicrobial resistance profile, and resistance genes of the 21 clinical *P. aeruginosa* strains, Table S2: MICs of doxycycline, minocycline, and compound **3** against PA01 and *P. aeruginosa* Tunisian clinical strains, Table S3: Dose-dependent effect of compound **3** to enhance doxycycline and minocycline activities against PA01 and *P. aeruginosa* Tunisian clinical strains, Figure S1: ^1^H NMR spectrum of **3**, Figure S2: ^13^C NMR spectrum of **3**.
