# Supplementary material for: Efficiency of a Tetracycline-Adjuvant Combination Against Multidrug Resistant Pseudomonas aeruginosa Tunisian Clinical Isolates
Source: Antibiotics (Basel). 2020 Dec 17;9(12):919. doi: 10.3390/antibiotics9120919 (PMC7766271; doi:10.3390/antibiotics9120919)
Supplement: Supplementary file 1 [file antibiotics-09-00919-s001.pdf]

Supplementary Materials

Article

Efficiency of a Tetracycline-Adjuvant Combination Against Multidrug Resistant *Pseudomonas aeruginosa* Tunisian Clinical Isolates

Azza Troudi [1,2], Meha Fethi [2], Mohamed Selim El Asli [3], Jean Michel Bolla [1], Naouel Klibi [2] and Jean Michel Brunel [1,*]

1 Aix Marseille Univ, INSERM, SSA, MCT, 13385 Marseille, France; azzatroudi.92@gmail.com (A.T.); jean-michel.bolla@univ-amu.fr (J.M.B.)

2 Laboratory of Microorganisms and Active Biomolecules, Department of Biology, Faculty of Sciences of Tunis, University of Tunis El Manar, Tunis, Tunisia; meha.fethi@yahoo.fr (M.F.); n_klibi@yahoo.fr (N.K.)

3 Service of Microbiology, Military Hospital of Tunis HMPIT, Tunis, Tunisia; elasliselim@yahoo.fr

* Correspondence: jean-michel.brunel@inserm.fr

General procedure for the synthesis of polyaminoisoprenyl derivatives 3

To a solution of spermine (450 mg, 2.27 mmol) and triethylamine (450 µL, 4.5 mmol) in distillated tetrahydrofurane (THF) (10 mL) is added dropwise farnesyl chloride **1** (mixture of isomers) (480 mg, 2 mmol) in distillated THF (15 mL). The reaction mixture is stirred at room temperature for 24 h. and evaporated to dryness. The crude residue is purified by column chromatography (eluant CH_2Cl_2/MeOH/conc.NH_4OH, 7:3:1) to afford the pure desired compound as a yellow solid in 64% yield (mixture of isomers).

Compound 3. 64% yield; Yellow solid; ^{1}H NMR (MeOD, 250 MHz): δ = 5.22-5.25 (m, 1H), 5.03-5.14 (m, 2H), 3.11-3.25 (m, 3H), 2.50-2.73 (m, 9H), 2.43-2.49 (m, 3H), 1.96-2.11 (m, 10H), 1.69-1.74 (m, 12H), 1.50-1.67 (m, 10H). ^{13}C (MeOD): δ = 140.06, 139.44, 136.28, 136.25, 136.16, 136.13, 132.37, 132.13, 132.10, 125.94, 125.42, 125.38, 125.21, 123.15, 121.80, 54.58, 52.48, 52.25, 50.66, 50.52, 48.77, 48.24, 48.01, 47.63, 41.28, 41.18, 41.12, 40.94, 40.90, 40.80, 40.62, 33.26, 32.99, 30.35, 30.11, 28.53, 28.21, 27.84, 27.66, 27.43, 26.09, 26.04, 25.41, 23.84, 23.79, 17.90, 17.86, 16.61, 16.48, 16.25, 16.21. $C_{25}H_{50}N_4$ MS (ESI+) m/z 407.41 (100%, $[M + H]^+$).

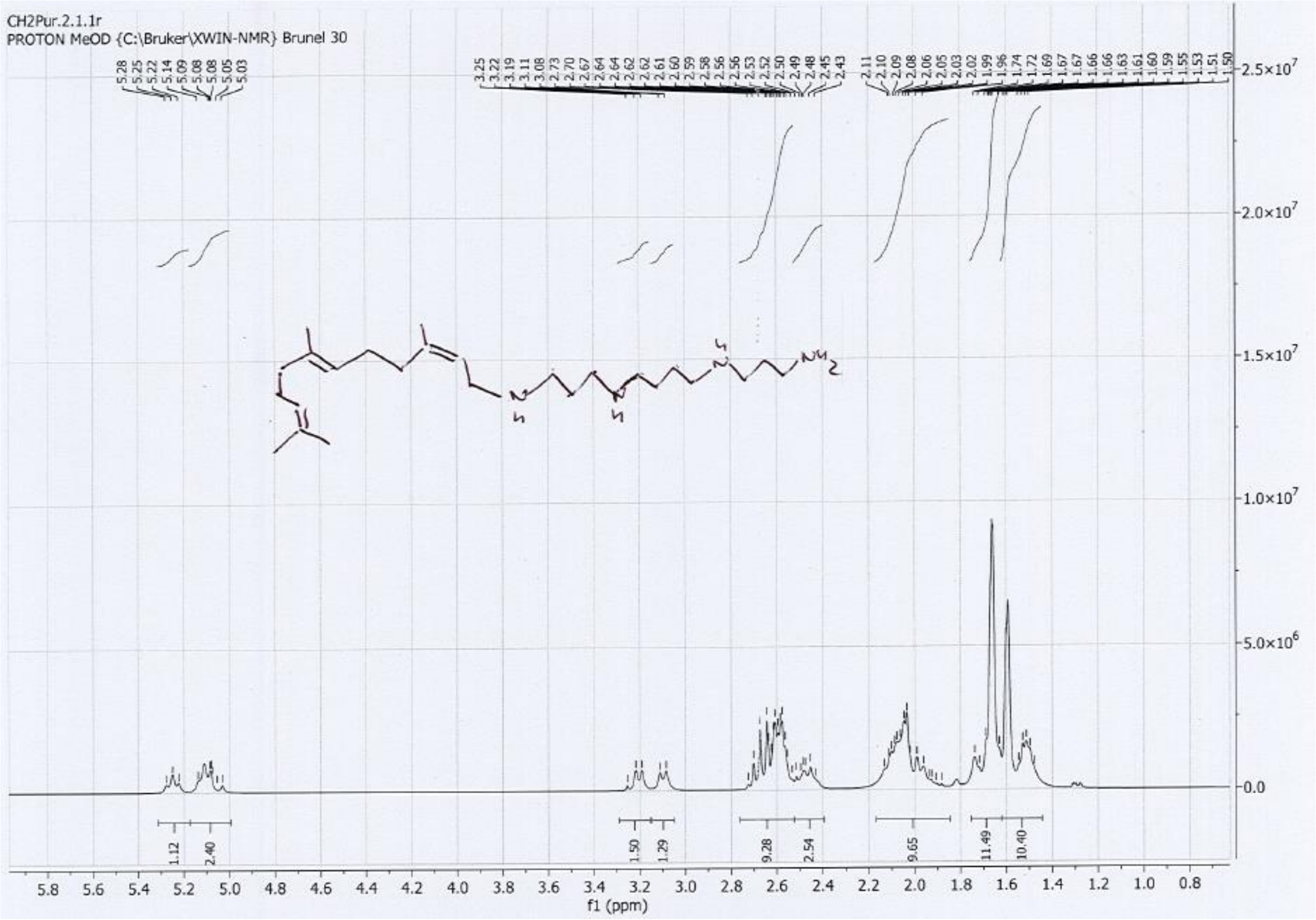

Figure S1. ^{1}H NMR spectrum of 3.

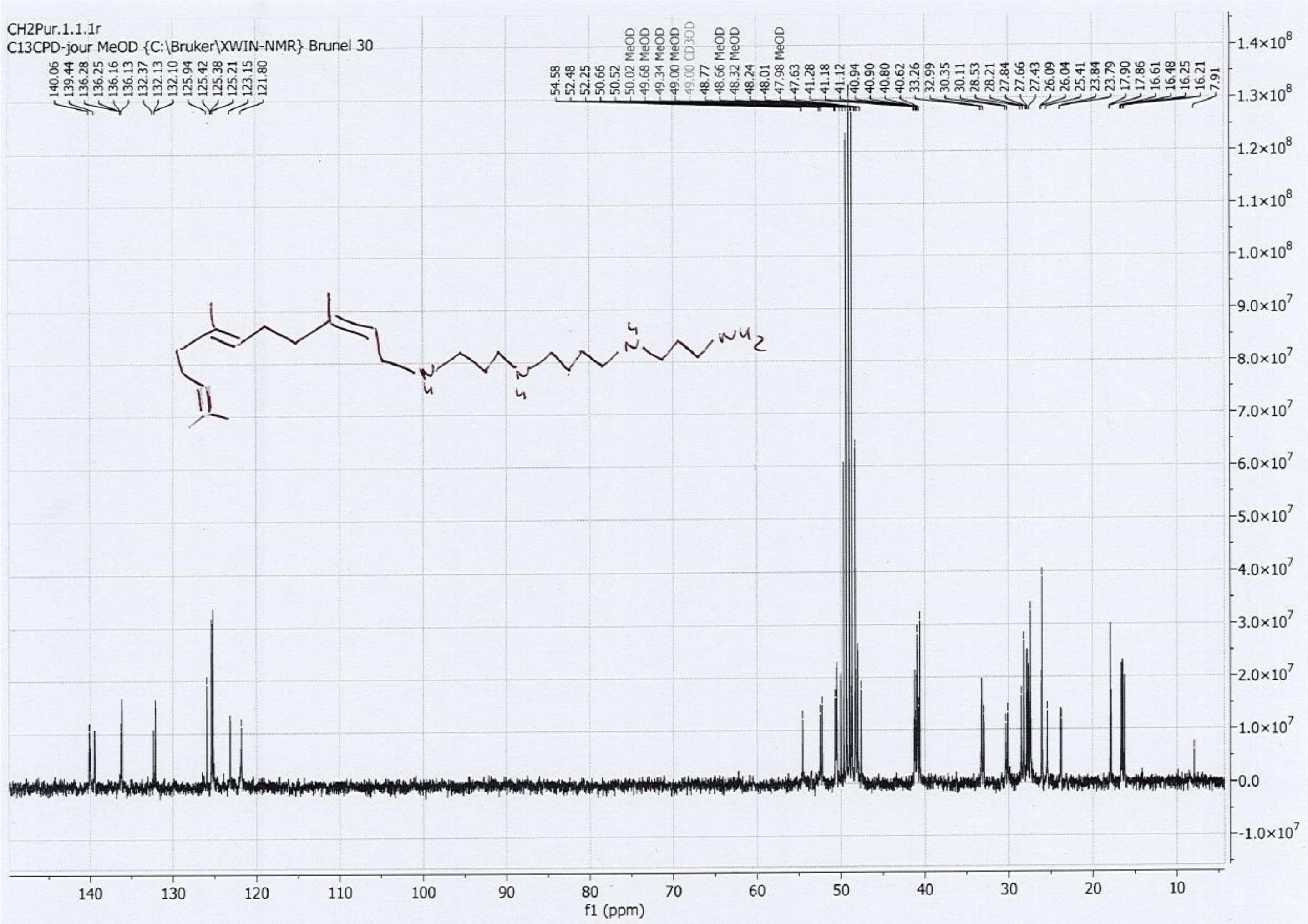

Figure S2. ^{13}C NMR spectrum of 3.

Table S1. Origins, antimicrobial resistance profile, and resistance genes of the 21 clinical *P. aeruginosa* strains.

Strain	Ward	Origin	Profil of resistance	Resistance genes	Efflux pump test
P1	ICU	blood	IMP, MEM, CAZ, FEP, TCC, PIP, PIT, AZT, AN, GM, TM, CIP, LVX	SHV	-
P2	ICU	tracheal aspirate fluid	IMP, MEM, CAZ, FEP, TCC, PIP, PIT, AZT, AN, GM, TM, CIP, LVX	VIM, GES	+
P3	ICU	catheter	IMP, MEM, CAZ, FEP, TCC, PIP, PIT, AZT, AN, GM, TM, CIP, LVX	SHV	+
P4	ICU	blood	IMP, MEM, CAZ, FEP, TCC, PIP, PIT, AZT, AN, GM, TM, CIP, LVX	VIM, GES	+
P6	orthopedic surgery	pus	IMP, MEM, CAZ, FEP, TCC, PIP, PIT, AZT, GM, TM, CIP, LVX	SHV	-
P8	ICU	tracheal aspirate fluid	IMP, MEM, CAZ, FEP, TCC, PIP, PIT, AZT AN, GM, TM, CIP, LVX	GES	+
P11	ICU	expectoration	IMP, MEM, CAZ, FEP, TCC, PIP, PIT, AZT, AN, GM, TM, CIP, LVX	SHV	+
P12	ICU	tracheal aspirate fluid	IMP, MEM, TCC, PIP, PIT, AZT, AN, GM, TM, CIP, LVX	GES	+
P26	vascular surgery	pus	IMP, MEM, FEP, TCC, PIP, PIT, TM, CIP, LVX	-	+
P28	ICU	tracheal aspirate fluid	IMP, MEM, FEP, TCC, PIP, PIT, AZT AN, GM, TM, CIP, LVX	GES	+
P29	ICU	catheter	IMP, MEM, FEP, TCC, PIP, PIT, AZT, AN, GM, TM, CIP, LVX	GES	+
P34	ICU	tracheal aspirate fluid	IMP, MEM, FEP, TCC, PIP, PIT, AZT, AN, GM, TM, CIP, LVX	GES	+
P45	ICU	blood	IMP, MEM, TCC, PIP, AZT		+
P50	ICU	tracheal aspirate fluid	IMP, MEM, FEP, TCC, PIP, PIT, AZT, AN, GM, TM, CIP, LVX	GES	+
P51	ICU	blood	IMP, MEM, FEP, TCC, PIP, AZT, AN, GM, TM, CIP, LVX	-	+
P54	ICU	blood	IMP, MEM, CAZ, FEP, TCC, PIP, PIT, AZT, GM, TM, CIP, LVX	VIM	+
P69	ICU	tracheal aspirate fluid	IMP, MEM, CAZ, FEP, TCC, PIP, PIT, AZT, AN, GM, TM, CIP, LVX	GES	+
P72	ICU	tracheal aspirate fluid	IMP, MEM, CAZ, FEP, TCC, PIP, PIT, AZT, AN, GM, TM, CIP, LVX	GES	+
P73	urology	urine	IMP, MEM, CAZ, FEP, TCC, PIP, AZT, GM, TM, CIP, LVX	GES	+
P74	bacteriology	tracheal aspirate fluid	IMP, MEM, CAZ, FEP, TCC, PIP, PIT, AZT, AN, GM, TM, CIP, LVX	SHV	+
P569	-	tracheal aspirate fluid	IMP, MEM, AZT, AN, GM, TM, LVX	GES	+

amikacin (AN), aztreonam (ATM), cefepime (FEP), ceftazidime (CAZ), ciprofloxacin (CIP), gentamicin (GEN), imipenem (IPM), levofloxacin (LVX), meropenem (MEM), piperacillin (PIP), piperacillin-tazobactam (PIT), ticarcillin-clavulanic acid (TCC), tobramycin (TM), Intensive care Unit (ICU).

Table S2. MICs of doxycycline, minocycline, and compound **3** against PA01 and *P. aeruginosa* Tunisian clinical strains.

Strains	MIC of doxycycline (μg/mL)	MIC of minocycline (μg/mL)	MIC of compound 3 (μM) (μg/mL)
**PA01**	64	32	25 (10)
**P1**	64	32	25 (10)
**P2**	64	32	25 (10)
**P3**	64	32	25 (10)
**P4**	64	32	25 (10)
**P6**	64	32	12.5 (5)
**P7**	128	128	25 (10)
**P8**	64	32	25 (10)
**P11**	128	64	25 (10)
**P12**	64	32	25 (10)
**P14**	64	32	25 (10)
**P26**	128	128	25 (10)
**P28**	64	64	25 (10)
**P29**	64	64	25 (10)
**P34**	64	64	25 (10)
**P45**	128	64	25 (10)
**P50**	64	64	25 (10)
**P51**	64	32	25 (10)
**P54**	64	32	25 (10)
**P69**	64	32	25 (10)
**P72**	64	32	25 (10)
**P73**	128	128	25 (10)
**P74**	128	64	50 (20)
**P569**	64	64	50 (20)

Table S3. Dose-dependent effect of compound **3** to enhance doxycycline and minocycline activities against PA01 and *P. aeruginosa* Tunisian clinical strains.

Strains	MIC of doxycycline in the presence of compound 3 at different concentrations			MIC of minocycline in the presence of compound 3 at different concentrations		
	2.5 µM	5 µM	10 µM	2.5 µM	5 µM	10 µM
			(Gain factor)			
**PA01**	8 (8)	2 (64)	0.5 (128)	4 (8)	1 (64)	0.25 (128)
**P1**	2 (32)	1 (64)	**<0.005 (>1024)**	2 (16)	0.5 (64)	**<0.005 (>1024)**
**P2**	4 (16)	1 (64)	0.25 (256)	4 (8)	1 (32)	0.25 (128)
**P3**	4 (16)	2 (32)	0.25 (256)	4 (8)	2 (16)	0.25 (128)
**P4**	4 (16)	2 (32)	0.25 (256)	8 (4)	1 (32)	0.25 (128)
**P6**	1 (64)	0.5 (128)	**<0.005 (>1024)**	1 (32)	0.25 (128)	**<0.005 (>1024)**
**P7**	4 (16)	2 (64)	0.25 (256)	4 (16)	2 (64)	0.25 (256)
**P8**	8 (16)	2 (32)	0.5 (256)	8 (8)	2 (16)	0.5 (128)
**P11**	8 (16)	2 (64)	0.5 (256)	8 (8)	2 (32)	0.5 (128)
**P12**	4 (16)	2 (32)	0.5 (128)	4 (8)	2 (16)	0.5 (64)
**P14**	8 (8)	2 (32)	1 (64)	8 (4)	1 (32)	1 (32)
**P26**	8 (16)	2 (64)	1 (128)	8 (16)	1 (128)	0.5 (256)
**P28**	8 (8)	2 (32)	1 (64)	4 (16)	1(64)	0.5 (128)
**P29**	4 (16)	1 (64)	0.125 **(512)**	4 (16)	1 (64)	0.125 **(512)**
**P34**	4 (16)	1 (64)	0.25 (256)	4 (16)	1 (64)	0.125 **(512)**
**P45**	8 (16)	2 (64)	1 (128)	4 (16)	2 (32)	0.5 (128)
**P50**	8 (8)	2 (32)	0.5 (128)	8 (8)	4 (16)	0.5 (128)
**P51**	4 (16)	1 (64)	0.25 (256)	8 (4)	2 (16)	0.25 (128)
**P54**	8 (8)	1 (64)	0.5 (128)	8 (4)	1 (32)	0.25 (128)
**P69**	8 (8)	1 (64)	0.125 **(512)**	8 (4)	1 (32)	0.125 (256)
**P72**	4 (16)	1 (64)	0.5 (128)	8 (4)	2 (16)	0,0031 **(1024)**
**P73**	64 (2)	16 (8)	8 (16)	32 (4)	8 (16)	2 (64)
**P74**	32 (4)	8 (16)	4 (32)	16 (4)	4 (16)	1 (64)
**P569**	16 (4)	2 (32)	0.5 (128)	16 (4)	8 (16)	0.5 (128)
